# Occurrence of zoonotic gastrointestinal parasites of rodents and the risk of human infection in different biomes of Brazil

**DOI:** 10.29374/2527-2179.bjvm113820

**Published:** 2021-03-23

**Authors:** Victor Fernando Santana Lima, Rafael Antonio Nascimento Ramos, Alessio Giannelli, Wagner Wesley Araújo Andrade, Irma Yaneth Torres López, Ingrid Carla do Nascimento Ramos, Laura Rinaldi, Giuseppe Cringoli, Leucio Câmara Alves

**Affiliations:** 1 Veterinarian, DSc., Unidade de Medicina Veterinária, Universidade Federal de Sergipe (UFS), Nossa Senhora da Glória, SE, Brazil; 2 Veterinarian, DSc., Laboratório de Parasitologia, Universidade Federal do Agreste de Pernambuco (UFAPE), Garanhuns, PE, Brazil; 3 Veterinarian, DSc., Vaxinano, Lille, France; 4 Veterinarian, MSc., Departamento de Medicina Veterinária, Universidade Federal Rural de Pernambuco (UFRPE), Recife, PE, Brazil; 5 Veterinarian, MSc., Universidad de El Salvador, San Salvador, El Salvador; 6 Agronomist, MSc., Laboratorio de Parasitologia, UFRPE, Recife, PE, Brazil; 7 Veterinarian, DSc., University of Naples Federico II, Napoli, Italy; 8 Veterinarian, DSc., University of Naples Federico II, Napoli, Italy; 9 Veterinarian, DSc., Departamento de Medicina Veterinária, UFRPE, Recife, PE, Brazil

**Keywords:** zoonosis, parasitological techniques, synanthropic animals, rodent-borne diseases, FLOTAC, zoonose, técnicas parasitológicas, animais sinantrópicos, doenças transmitidas por roedores, FLOTAC

## Abstract

Rodents are synanthropic mammals adapted to several ecosystems, where they can contribute to the transmission of zoonotic pathogens, including gastrointestinal parasites. The aim of this study was to study the occurrence of gastrointestinal parasites from rodents and discuss the risk of transmission to humans. Fecal samples (n = 110) from different rodent species, namely, *Cerradomys subflavus* (n = 4), *Mus musculus* (n = 14), *Rattus norvegicus* (n = 80), *Rattus rattus* (n = 8) and *Thrichomys apereoides* (n = 4), were analyzed using the FLOTAC technique. Of the samples examined, 73.6% (81/110) tested positive for at least one gastrointestinal parasite. The most commonly identified parasites were *Aspiculuris* sp., *Hymenolepis nana*, *Moniliformis* sp., *Syphacia* sp., *Strongyloides* spp., *Taenia* spp*.,* and *Trichuris* spp. eggs, *Angiostrongylus cantonensis* larvae and *Entamoeba* spp. cysts. The findings of this study demonstrate that rodents living in different Brazilian biomes are parasitized by a wide range of parasites, including some of public health concern. Therefore, the proximity of rodents to human settlements may represent a tangible risk of infection for people living in these areas.

## Resumo

Roedores são mamíferos sinantrópicos adaptados a vários ecossistemas, onde podem contribuir para a transmissão de patógenos zoonóticos, incluindo parasitas gastrointestinais. O objetivo deste estudo foi descrever a ocorrência de parasitos gastrointestinais de roedores e discutir o risco de transmissão ao ser humano. Amostras fecais (n = 110) de diferentes espécies de roedores, a saber, *Cerradomys subflavus* (n = 4), *Mus musculus* (n = 14), *Rattus norvegicus* (n = 80), *Rattus rattus* (n = 8) e *Thrichomys apereoides* (n = 4), foram analisados pela técnica FLOTAC. Das amostras examinadas, 73,6% (81/110) apresentaram resultado positivo para pelo menos um parasito gastrointestinal. Os parasitos mais comumente identificados foram ovos de *Aspiculuris* sp., *Hymenolepis nana*, *Moniliformis* sp., *Syphacia* sp., *Strongyloides* spp., *Taenia* spp., *Trichuris* spp., larvas de *Angiostrongylus cantonensis* e cistos de *Entamoeba* spp. Os resultados deste estudo demonstraram que os roedores que vivem em diferentes biomas brasileiros são parasitados por uma ampla variedade de parasitos, incluindo alguns problemas de saúde pública. Portanto, a proximidade de roedores com assentamentos humanos pode representar um risco de infecção nessas áreas.

## Introduction

The order Rodentia includes about 40% of currently known mammalian species. During their evolution, these small animals have been able to adapt to different environments and to spread throughout the world over time (RatZooMan, 2006). In some cases, rodents have acclimatized so well to synanthropic biomes that they have become an integral part of the environment, thus participating in the life cycle of other animal species. Hence, through either symbiosis or mutualism, rodents can contribute to the spread of parasites of veterinary and medical concern (Rosický, 1978). The term “rodent-borne diseases” has been coined to describe a specific category of diseases, for which rodents act as the main reservoirs of life-threatening pathogens and conditions, such as Lassa fever, Leptospirosis, Plague and gastrointestinal parasites (Ogunniyi et al., 2014; Sumangali et al., 2012).

In terms of zoonotic parasites, their epidemiology shows various modes of transmission (Han et al., 2015). In most cases, rodents show no clinical signs of the presence of some parasites (e.g., *Angiostrongylus cantonensis*, *Hymenolepis diminuta*, *Giardia* spp. and *Cryptosporidium* spp.), which are highly harmful to humans. In this case scenario, rodents play a pivotal role as disseminators of these pathogens (Costa & Catto, 1994; Garedaghi & Khaki, 2014; Hamrick et al., 1990; Kulasiri, 1954; Reginatto et al., 2008; Vitta et al., 2011). Landscape fragmentation and urbanization have long been known to favor the parasitism of rodents by zoonotic gastrointestinal parasites (Jittapalapong et al., 2009; Karesh et al., 2012). In fact, many rodent species exhibit synanthropic behavior and often live in close contact with humans. This close interaction has favored the transmission of parasites between these hosts (Hamrick et al., 1990; Paramasvaran et al., 2009; Vitta et al., 2011).

Very few studies have so far been conducted to elucidate the mechanisms for transmission of rodent-borne parasites. This can be attributed mostly to the difficulty in collecting reliable samples, and to the low sensitivity of some diagnostic tools (Alves et al., 2007; Santos et al., 2015). Therefore, the aim of this study was to detect gastrointestinal parasites in wild and synanthropic rodents from different biomes in Brazil. The risk for infection of humans that share the same ecological niches with these rodents is also discussed.

## Material and methods

### Study area and ethical aspects

This study was conducted from March 2016 to September 2017 in nine different municipalities in the state of Pernambuco, northeastern Brazil (Table 1). These municipalities were situated in two distinct biomes: i) Caatinga, and ii) Atlantic Forest. The Caatinga biome, which is characterized by low trees and a dry environment, is home to a wide diversity of animals. Conversely, the Atlantic forest, which is considered Brazil’s most threatened biome, is characterized by the presence of tall trees (e.g., *Caesalpinia leiostachya*, *Cordia superba* and *Eugenia uniflora*) and mangroves.

**Table 1:** Localities per landscape type, mesoregion, their coordinates, sizes, climate and the species of rodents captured per locality in Pernambuco, Brazil.

Locality	Biome	Mesoregion	Geographic location	Size [km^2^]	Climate	Species of rodents captured
Barreiros	Atlantic forest	Forest Zone	08° 49’ 04” S, 35° 11’ 09” W	233,4	Humid tropical	*Rattus norvegicus*
Bodocó	Caatinga	Backwoods	07º 46’ 42” S, 39º 56’ 28” W	1.616,502	Tropical semi-arid	*Rattus rattus* and *Thrichomys apereoides*
Camaragibe	Atlantic forest	Metropolitan region	08º 01’ 18” S, 34º 58’ 52” W	51,257	Humid tropical	*Mus musculus*, *Rattus norvegicus* and *Rattus rattus*
Carnaíba	Caatinga	Backwoods	07° 48’ 18” S, 37° 47’ 38” W	437	Tropical semi-arid	*Rattus norvegicus*
Flores	Caatinga	Backwoods	07° 51’ 57” S, 37° 58’ 30” W	1.011	Tropical semi-arid	*Rattus norvegicus*
Gravatá	Caatinga and Atlantic forest	Agreste	08º 12’ 04” S, 35º 33’ 53” W	506,785	Tropical semi-arid and humid tropical	*Thrichomys apereoides*
Ilha de Itamaracá	Atlantic forest	Metropolitan region	07º 45’ 00” S, 34° 51’ 00” W	66.684	Humid tropical	*Cerradomys subflavus* and *Thrichomys apereoides*
Olinda	Atlantic forest	Metropolitan region	08º 00’ 32” S, 34º 51’ 19” W	41,681	Humid tropical	*Rattus norvegicus*
Recife	Atlantic forest	Metropolitan region	08º 03’ 14” S, 34º 52’ 52” W	218,435	Humid tropical	*Mus musculus*, *Rattus norvegicus* and *Rattus rattus*

All the procedures carried out in this study were approved by the Ethics Committee on Animal Use (CEUA) of the Federal Rural University of Pernambuco (Protocol no. 127/2015) and by Brazil’s Biodiversity Authorization and Information System (SISBIO), under Protocol no. 50588-1.

### Sampling and laboratory procedures

Rodents were captured using Tomahawk Live Traps (30 x 17.5 x 15.5 cm) baited with pineapple and peanut butter. The traps (n = 50) were placed at 6:00 pm and removed at 6:00 am, totaling an effort of 50 traps/night, and 250 trap/nights in five days of capture. Fecal samples were collected from wild (n = 8) and synanthropic (n = 102) rodents of different species [i.e., *Cerradomys subflavus* (n = 4), *Mus musculus* (n = 14), *Rattus norvegicus* (n = 80), *Rattus rattus* (n = 8) and *Thrichomys apereoides* (n = 4)] after spontaneous defecation. These samples were stored in plastic vials containing 10% formalin until laboratory processing. Using identification keys, all the captured rodents were identified, estimated age and taxonomically classified down to genus based on external characteristics (e.g., body size, weight and coat) (Bonvicino et al., 2008).

Lastly, the samples were processed individually by the FLOTAC technique, using two flotation solutions (sodium chloride, specific gravity, s.g. = 1.200 and zinc sulfate, s.g. = 1.350) (Cringoli et al., 2010). All the parasite stages observed in this study were identified based on previously described morphological features and taxonomic keys (Bowman et al., 2010; Taylor et al., 2017).

### Data analysis

The data were statistically analyzed to ascertain absolute and relative frequency. The Chi-Square with Yates correction (x^2^) was used to compare positivity between different sexes, ages and origins, using a significance level of 5%. All the analyses were performed using BioEstat version 5.0 statistical software (Ayres et al., 2007).

## Results

Overall, 73.6% (81/110) of the samples tested positive for at least one gastrointestinal parasite (e.g., cysts, eggs and/or larvae). Moreover, 9.8% of the fecal samples (8/81) were collected from young rodents and 90.2% (73/81) from adults (x^2^ = 19.68; p = 0.0000); 38.2% (31/81) came from females and 61.8% (50/81) from males (x^2^ = 0.884; p = 0.4714). In addition, 10.7% (8/81) and 89.3% (73/81) were collected from animals inhabiting areas of Caatinga and Atlantic Forest (x^2^ = 19.68; p = 0.0000), respectively (Table 2). Six different genera of endoparasites were observed in wild (57.2%) and synanthropic (42.8%) rodents from the Caatinga biome (p = 0.0000).

**Table 2:** Frequency of zoonotic gastrointestinal parasites in the fecal samples, according to class, genus or species of parasites and rodent species.

Class	Genus/Specie	Cerradomys subflavus*^a^	Number of positive samples of rodent specie				Frequency % (n/N)
			Mus musculus^b^	Rattus norvegicus^b^	Rattus rattus^c^	Thrichomys apereoides*^c^	
Archiacanthocephala	*Moniliformis* sp.	^-^	^-^	-	2/8	^-^	1.81 (2/110)
Cestoda	*Hymenolepis nana*	1/4	^-^	9/80	^-^	^-^	9.09 (10/110)
	*Taenia* spp.	^-^	^-^	4/80	^-^	^-^	3.63 (4/110)
Nematoda	*Angiostrongylus cantonensis*	^-^	^-^	14/80	^-^	^-^	12.72 (14/110)
	*Aspiculuris* sp.	^-^	^-^	-	4/8	^-^	3.63 (4/110)
	*Strongyloides* spp.	1/4	3/14	34/80	1/8	^-^	35.45 (39/110)
	*Syphacia* sp.	^-^	^-^	5/80	^-^	^-^	4.54 (5/110)
	*Trichuris* spp.	2/4	^-^	2/80	^-^	4/4	7.27 (8/110)
Protozoa	*Entamoeba* spp.	^-^	^-^	2/80	4/8	^-^	5.45 (6/110)
Number of rodent		4	14	80	8	4	110

Note: *Native species; ^a^Biome of Caatinga; ^b^Biome of Atlantic forest; ^c^Both biomes; ^-^Parasitism absence.

Among the positive animals, 71.4% and 28.6% (x^2^ = 18.68; p = 0.0000) were rodents that inhabit forest and urban areas, respectively. In addition, a total of nine different genera of gastrointestinal parasites were identified in synanthropic rodents living in the domiciliary and peridomiciliary area of the metropolitan region of Recife (Atlantic Forest biome).

**Figure 1:**
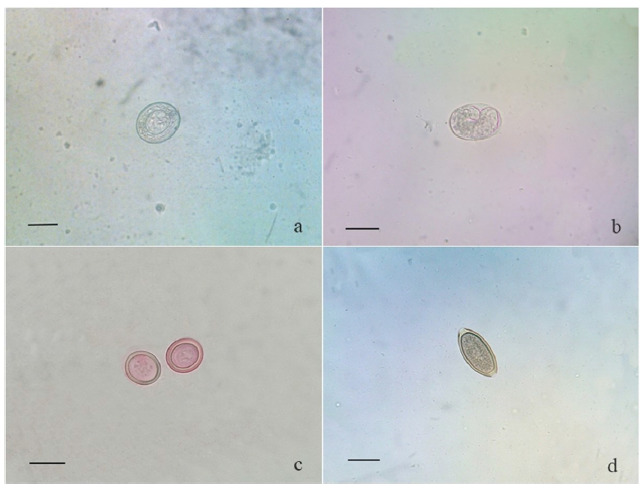
Helminth and cestode eggs detected by FLOTAC in wild and synanthropic rodents in Brazil (Scale bar = 25 μm). (a) *Hymenolepis* nana: oval egg, 40-60 μm long and 30-50 μm wide, containing oncospheral hooks and polar filaments within the space between the oncosphere and outer shell; (b) *Strongyloides* spp.: thin-shelled oval egg, 40-50 μm long and 30-34 μm wide. (c) *Taenia* spp.: oval egg, 30-40 μm long and 30-40 μm wide, containing a hexacanth embryo; (d) *Trichuris* spp.: bar-shaped colorless thick-shelled egg, 48-62 μm long and 29-37 μm wide, with bipolar plugs, with 400x objective in optical microscopy.

Infection rates with acanthocephalans, cestodes, nematodes and protozoa were 3.1%, 12.2%, 75.8%, and 9.1%, respectively. The parasites most frequently detected were *Strongyloides* spp. (35.45%; 39/110) and *Hymenolepis nana* (9.09%; 10/110; Figure 1a) eggs, *Angiostrongylus cantonensis* (12.72%; 14/110) larvae and *Entamoeba* spp. (5.45%; 6/110) cysts. As for parasite loads, *Strongyloides* spp. (up to 10,609 eggs; Figure 1b), *H. nana* (up to 5,652 eggs), *A. cantonensis* (up to 3,899 larvae) and *Entamoeba* spp. (up to 2,124 cysts) were the most abundant.

Coinfections were found in 65.2% (52/81) of the samples, the most common one being simultaneous infection with *A. cantonensis* larvae and *Strongyloides* spp. eggs (25%; 13/52).

The largest number of positive samples (87.8%; 71/81) for cysts, eggs and/or larvae of gastrointestinal parasites was found in synanthropic rodents, with eggs of *Syphacia* sp., *Taenia* spp. (Figure 1c) and larvae of *A. cantonensis* detected only in *R. norvegicus*. *Aspiculuris* sp. and *Moniliformis* sp. eggs were identified only in fecal samples from *R. rattus*. *Trichuris* sp. (Figure 1d) was detected in fecal samples of *R. norvegicus* and *T. apereoides*.

## Discussion

The findings of this study indicate that synanthropic and wild rodents from Caatinga and Atlantic Forest biomes in Brazil are parasitized by a wide range of gastrointestinal parasite species of public health concern. These findings suggest that landscape features can affect the epidemiology of zoonotic parasites in rodents.

The prevalence data garnered in this study were much higher than those reported in previous surveys conducted in Iran-IRN (Ahmad et al., 2014), Pakistan-PAK (Garedaghi & Khaki, 2014) and Italy-ITA (d’ovidio et al., 2015), which involved the analysis of rodent fecal samples using the traditional spontaneous sedimentation technique, centrifugal flotation and the FLOTAC method, which reported positivity rates of 13.9% (500/3,600), 35% (19/57) and 57% (24/172), respectively.

Among the gastrointestinal pathogens identified in this study, it should be pointed out that the infective stages of parasites of the genera Angiostrongylus, Entamoeba, Hymenolepis, Moniliformis, Strongyloides, *Syphacia*, *Taenia* and *Trichuris* have a proven zoonotic potential (Aghazadeh et al., 2015; Angal et al., 2015; Ashford & Crewe, 2003; Miyazak, 1991). Hence, the presence of these animals in areas inhabited by humans in Brazil represents a public health risk (Paramasvaran et al., 2009), in view of possible environmental contamination by rodent feces and the parasites they carry.

For *Angiostrongylus cantonensis*, in particular, causes eosinophilic meningoencephalitis, and this disease has already been identified in Brazil (Caldeira et al., 2007), as do cestodes such as *Hymenolepis nana* and *Taenia* spp. (Huggins et al., 1993). In this study, *A. cantonensis* larvae were detected in fecal samples from *R. norvegicus*, which is considered the definitive natural host. The life cycle of this nematode is generally shared among snails of the species *Achatina fulica, Helix aspersa* and *Helix pomatia* as intermediate host, and rodents (Lima et al., 2009; Oliveira et al., 2015; Vitta et al., 2011). Humans are considered accidental hosts infected through the ingestion of third-stage larvae, which, once inside the accidental host, induce acute disease that may even culminate in death or permanent disability (Morassutti et al., 2014). Similarly, *H. nana* and *Taenia* spp. are helminths that parasitize humans and may cause diarrhea, abdominal pain, irritability, and weight loss (Galan-Puchades, 2015; Muehlenbachs et al., 2015).

On the other hand, parasites of the genus *Syphacia* cause asymptomatic infection in their hosts, due to their low pathogenicity or the high degree of host/parasite adaptation. Parasitism of humans by the genus *Syphacia* is extremely rare, with only a few reports in the United States and Philippines (Pereira, 2009). *Moniliformis moniliformis* has frequently been detected based on the identification of adult parasites during necropsy examination (Lim et al., 1974). However, in this study, the infection was identified based on the detection of eggs in fecal samples of *R. rattus.* Cases of humans infected by this cestode have been described in Japan, where the infection was attributed to the close proximity of infected *R. norvegicus* to dwellings (Miyazak, 1991). With regard to amoebas, the genus *Entamoeba* is commonly found in rodents (i.e., *R. norvegicus* and *R. rattus*), but a growing number of deaths among human patients have been reported due to this protozoan genus (Lau et al., 2014; Nateghpour et al., 2014; Silva et al., 2014).

In this study, wild rodents from the Caatinga biome were found to carry a smaller variety of gastrointestinal parasites than those from the Atlantic Forest. This finding may be attributed to the high density of human dwellings and factories, which also represent highly populated urban centers (e.g., Camaragibe, Itamaracá, Recife and Olinda). These environments provide optimal conditions for the emergence of several species of synanthropic animals, such as rodents or arthropods (Vasconcelos et al., 2012).

It is important to note that helminth infections may be attributed to the susceptibility of some synanthropic animals, their behavior and immunological status, and environmental contamination (Anderson & Gordon, 1982; Scott & Gibbs, 1986). In this context, it has been proven that rodents living in urban areas are potential reservoirs of various species of gastrointestinal parasites (Sumangali et al., 2012). Most likely, urbanization plays an important role in the dissemination of these pathogens, given the close interaction among rodents, domestic animals and humans. The higher frequency of parasites in male (61.8%) than in female (38.2%) rodents indicates that the parasite load in these animals probably depends upon sex, and that males usually roam over a wider area in search of food (Ahmad et al., 2014; Ozanan, 1969). Moreover, parasitism is more common in adult rodents than in young animals, probably due to their roaming behavior and the pre-patent period (Stojcevic et al., 2004).

A noteworthy fact is that this is the first time *Aspiculuris* sp., *Moniliformis* sp., *Taenia* spp., and *Trichuris* spp. eggs, *A. cantonensis* larvae, and *Entamoeba* spp. cysts were detected in fecal samples from *C. subflavus, M. musculus, R. norvegicus, R. rattus* and *T. apereoides* in Brazil using the FLOTAC technique.

## Conclusion

The findings of this study indicate that a large diversity of zoonotic helminths and protozoa of public health importance can be detected in rodents from Brazil’s Caatinga and Atlantic Forest biomes.
